# Methods to mitigate *Escherichia coli* blooms in human *ex vivo* colon model experiments using the high throughput micro-Matrix bioreactor fermentation system

**DOI:** 10.1016/j.mex.2023.102393

**Published:** 2023-10-06

**Authors:** Harsh Mathur, Monica A. Mechoud, Chloe Matthews, Cathy Lordan, Jamie A. FitzGerald, Tom Beresford, Paul D. Cotter

**Affiliations:** a Teagasc Food Research Centre, Moorepark, Fermoy, County Cork, Ireland; b Food for Health Ireland, Teagasc Food Research Centre, Moorepark, Fermoy, County Cork, Ireland; c APC Microbiome Ireland, University College Cork, Cork, Ireland

**Keywords:** *Escherichia coli*, Gut microbiome, Blooms, Metagenomics, Optimised step-by-step methods to attenuate *Escherichia coli* blooms using the micro-Matrix bioreactor fermentation platform as an *ex vivo* model of the human distal colon

## Abstract

*Ex vivo* colon model experiments are frequently employed as a means to assess the gut microbiome modulating potential of different foods, food ingredients and dietary supplements. A number of useful models already exist; however, they tend to be relatively low in terms of throughput (3–4 samples per experiment) with a long experiment duration of one to a number of weeks. Therefore, a need for a high-throughput system with a short duration time is required to enable screening of large numbers of samples. Therefore, we report here on the development of a system based on the Applikon micro-Matrix bioreactor which has the capacity to run 24 samples with an experiment duration of 48 h. However, *Escherichia coli* blooms are a common problem encountered in this model. Here, we describe the factors that contribute to such blooms and provide approaches to address them, providing:

• Step by step optimisation of processes involved in conducting *ex vivo* distal colon experiments using the micro-Matrix bioreactor fermentation platform
• Recommended steps for users on how to attenuate *E. coli* blooms in such *ex vivo* colon model experiments.

## Graphical abstract

Flow chart depicting the main steps involved in conducting ex vivo colon model experiments.

**Figure:**
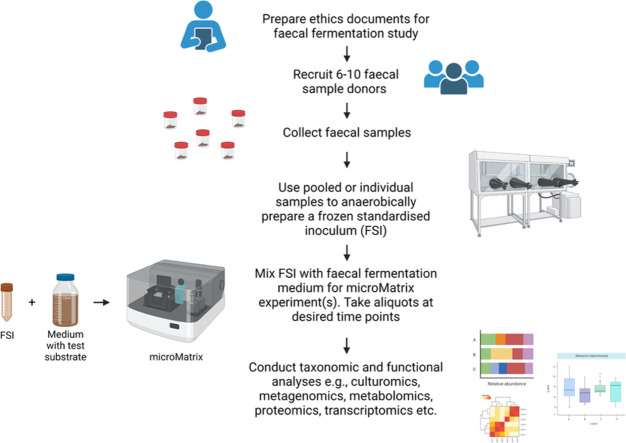

Specifications table

**Table:** 

Subject area:	Immunology and Microbiology
More specific subject area:	Human gut microbiome
Name of your method:	Optimised step-by-step methods to attenuate *Escherichia coli* blooms using the micro-Matrix bioreactor fermentation platform as an *ex vivo* model of the human distal colon.
Name and reference of original method:	Minor optimisation of methods involved in preparing frozen standardised inoculum (FSI) faecal slurry described in O'Donnell et al. 2016 [1] and conducting *ex vivo* colon model experiments using the micro-Matrix bioreactor fermentation system described in O'Donnell et al. 2018 [2] with a specific focus on attenuating *E. coli* blooms in such experiments.
Resource availability:	Equipment includes the micro-Matrix bioreactor fermentation system (Applikon). The data for this study have been deposited in the European Nucleotide Archive (ENA) at EMBL-EBI under accession number PRJEB64001 (https://www.ebi.ac.uk/ena/browser/view/PRJEB64001).
	

## Method details

**Fig. 1:**
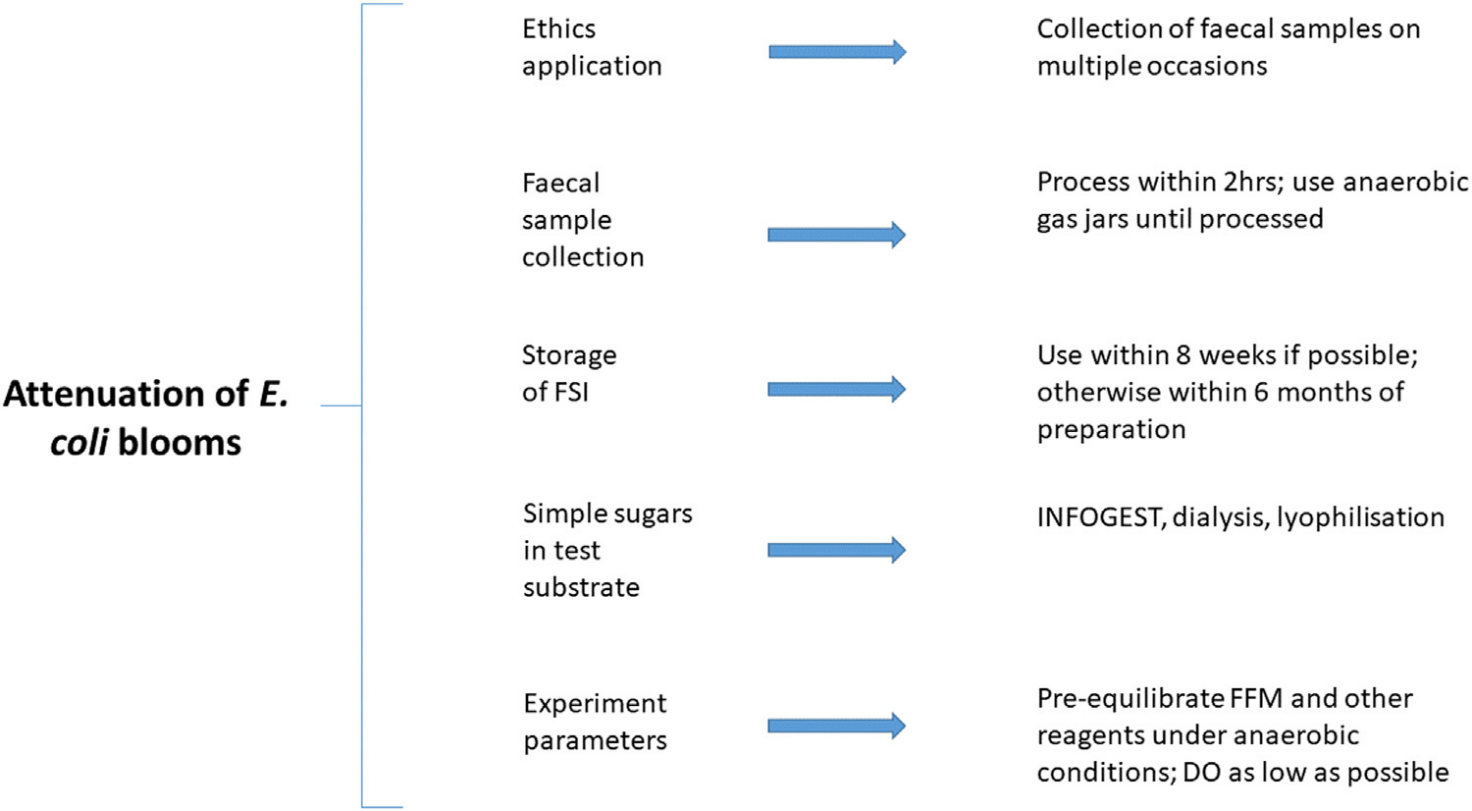
Flow chart depicting the main steps involved in *ex vivo* colon model experiments where our recommended optimisations are critical to mitigate *E. coli* blooms.

Experiments involving the use of *ex vivo* colon models are regularly conducted with a view to assessing the impact of a variety of substrates, nutrients, drugs, antimicrobials, amongst other substances on the gut microbiome. The micro-Matrix bioreactor fermentation system (Applikon) is an example of a platform that can be employed to undertake such experiments. However, researchers using this system often encounter *Escherichia coli* blooms. Herein we report the complex combination of factors that contribute to *E. coli* blooms in such experiments. More specifically, we list these causative factors, provide evidence to support their description as causative factors, and share insights gained from our troubleshooting efforts with a view to attenuating such blooms. Examples of such factors include the prolonged storage of the frozen standardised inoculum (FSI) faecal slurry at −80 °C, the presence of simple sugars such as glucose in the substrates tested, the broader composition of the substrates/samples tested as well as the concentration of dissolved oxygen (DO) present during the *ex vivo* colon model experiment. Overall, we describe and outline revised optimised standard operating procedures (SOPs) and appropriate steps with a view to attenuating *E. coli* blooms (Fig. 1), specifically when using the micro-Matrix bioreactor fermentation platform as an *ex vivo* model of the human distal colon.

**1. Recommended optimised method for frozen standardised inoculum (FSI) preparation (derived and adapted from O'Donnell et al. 2016)** [1] **), to minimise downstream *E. coli* blooms:**

○ Seek ethical approval to collect faecal samples from a cohort of 6–10 volunteers. We strongly recommend seeking approval to collect faecal samples on multiple occasions to avoid the need to store FSI for prolonged periods.
○ Collect faecal samples in sterile faecal sample pots and store at 4 °C (ideally not for more than 3 hrs) until all samples from the 6–10 volunteers are collected and ready to be processed together. To minimise the loss of viability of strict obligate anaerobes present in the faecal samples after they are excreted, sample pots should be stored in anaerobic gas jars with anaerobic gas packs (examples of which include Anaerocult^R^ A gas packs) at 4 °C until they are ready for processing.
○ Once all the samples are collected, immediately place the faecal pots of samples in an anaerobic chamber at 37 °C for pooling and processing.
○ Carefully weigh out equal quantities of each faecal sample using sterile spatulas from each of the volunteers until a total of 400 g of pooled faecal matter is attained. For example, if 10 volunteers have donated samples, weigh out 40 g from each sample.
○ Place the pooled faeces into a sterile stomacher bag which contains a sieve (examples of which include Seward Stomacher^R^ bags) in the anaerobic chamber being used to process the samples.
○ Carefully add 400 ml of 50 mM phosphate buffer (with 0.05% w/v L-cysteine hydrochloride) to the stomacher bag containing the pooled faeces, and mix together by hand for 2 min to ensure that a homogenous mix is attained. The solid faecal matter and liquid faecal slurry are then separated using the sieve in the stomacher bag to retain the solid material while allowing the liquid slurry to be decanted into 50 ml conical falcon tubes.
○ Weigh and balance the falcon tubes and centrifuge at 4000*g* for 25 min at ambient room temperature (approximately 18 °C).
○ Reconstitute the pellets, containing the faecal microbiota in the falcon tubes to the original starting volume with sterile 50 mM phosphate buffer (with 0.05% w/v L-cysteine hydrochloride) containing 25 % (v/v) glycerol.
○ Pool the reconstituted faecal microbiota suspensions from the falcon tubes into a sterile bottle.
○ Carefully mix the pooled microbiota suspension by inverting the bottle 3–4 times.
○ Aliquot 12 ml of the homogenous pooled faecal microbiota suspension into several 15 ml falcon tubes.
○ Store the aliquots at −80 °C. These are now referred to as ‘frozen standardised inoculum’ (FSI).
○ One sample of FSI is used for each downstream micro-Matrix *ex vivo* colon model experiment.

**2. Recommended optimised method for *ex vivo* colon model experiments using the micro-Matrix bioreactor fermentation system for testing liquid substrates or reconstituted solid substrates mixed in a 1:1 ratio with the Faecal Fermentation Medium (FFM)/Frozen Standardised Inoculum (FSI) mix, used as an example below (derived and adapted from O'Donnell et al. 2018** [2] **and guidelines in Applikon manuals, white papers and brochures). We advise users to perform calculations relating to the volumes and concentrations of FFM required, depending on specific needs and experimental design:**

**Fig. 2:**
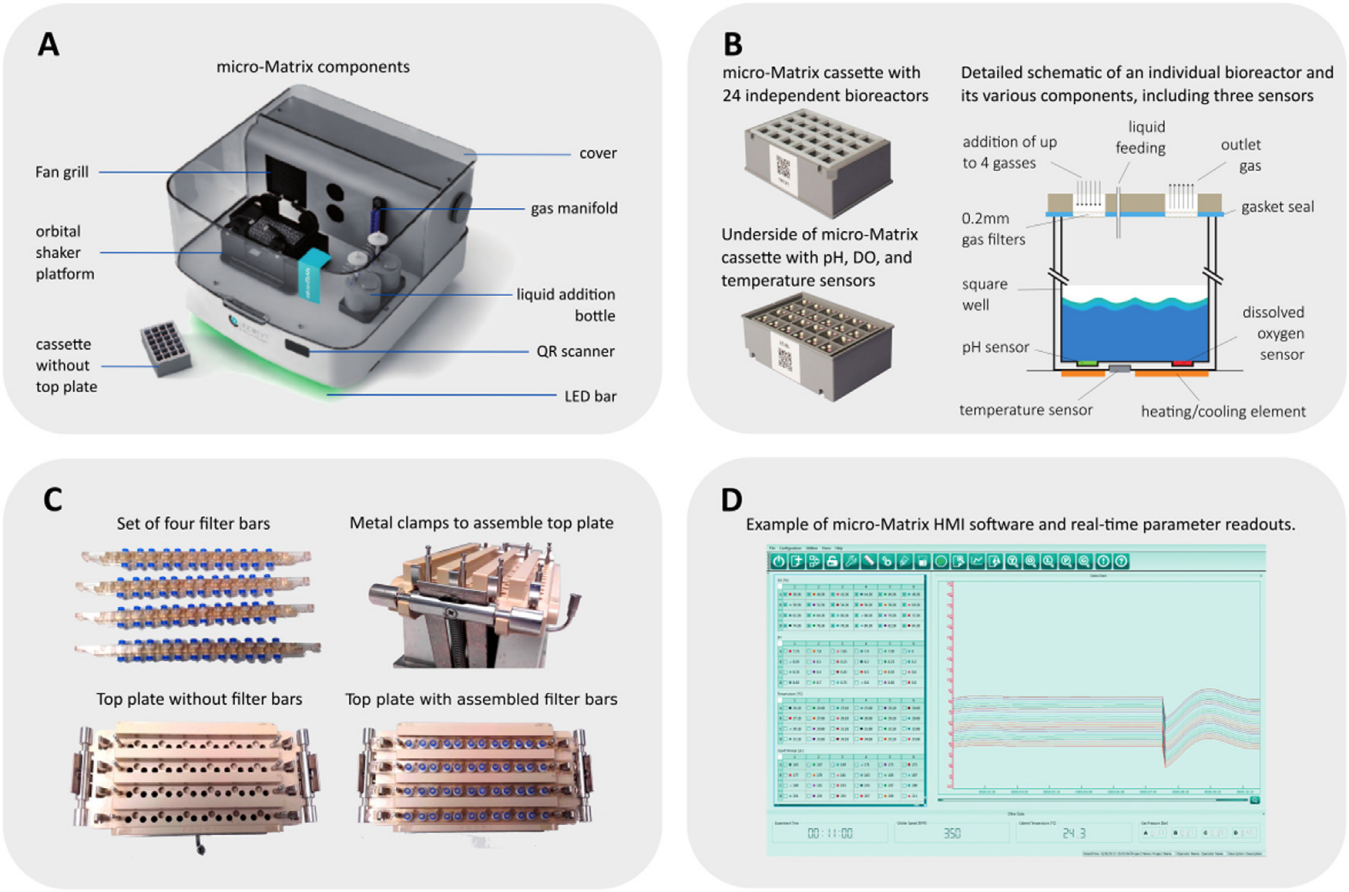
A) Image depicting the main components of the micro-Matrix system; B) Image depicting the design of a micro-Matrix cassette including a schematic of an independent bioreactor, consisting of the pH, DO and temperature sensors; C) Images depicting the filter bars, clamps and top plate used as part of the micro-Matrix system; D) Image depicting an example of the real-time parameters raw data readouts from the user-friendly HMI software. Figures depicting the sensors and fermentation unit derived from Applikon micro-Matrix brochure, white papers and product websites including Antteknik and Intekgroup. https://www.antteknik.com/en/products/?p=micro-matrix-bioreactors-1–10-ml. https://intekgroup.com.co/wp-content/uploads/2021/02/catalogo-microbiorreactores.pdf.
○ Prior to commencing the micro-Matrix experiment, autoclave the micro-Matrix top plate in an autoclave bag using a 121 °C for 15 min cycle (images depicted in Fig. 2). New pre-sealed sterile filter bars can be used for each experiment. However, if filter bars need to be re-used, autoclave filter bars in a beaker covered with tin foil using a 121 °C for 15 min cycle. Immediately after autoclaving, dry the micro-Matrix top plate and filter bars in a laminar flow hood for 2 hrs until all residual moisture is removed. Residual moisture can prevent the formation of a perfectly hermetic seal when assembling filter bars onto the top plate and thus, it is essential that both the top plate, as well as the four filter bars are completely dry.
○ Assemble filter bars carefully onto the top plate under aseptic conditions in a laminar flow hood. If required, inspect the underside of the top plate to ensure that perfectly hermetic seals are formed with the filter bars. A perfectly hermetic seal will ensure that the system remains aseptic, as well as ensuring that completely anaerobic conditions are maintained throughout the micro-Matrix *ex vivo* colon model experiment, by preventing the introduction of oxygen from the environment.
○ Pre-equilibrate all plastic-ware, other materials and reagents in an anaerobic chamber overnight prior to commencing the micro-Matrix experiment.
○ Prior to commencing the preparation of the micro-Matrix cassette, ensure that the BIP^R^ nitrogen and CO_2_ cylinders supplying the micro-Matrix unit have sufficient gas supplies for the entire duration of the experiment. BIP^R^ nitrogen is a higher quality grade of nitrogen that is recommended for micro-Matrix units.
○ Ensure that the liquid ammonia (20 % v/v), used for pH control is not more than four weeks old and that there is a sufficient volume for the duration of the experiment.
○ Introduce substrates/samples to be tested into the anaerobic chamber 2 hrs prior to commencing the experiment in order to pre-equilibrate under strict anaerobic conditions. Approximately 2 hrs of pre-incubation is sufficient for substrates/samples which contain live microbes and/or are prone to microbial contamination. However, substrates/samples which do not contain live microbes and those which are not amenable to microbial contamination can be pre-incubated under anaerobic conditions overnight prior to commencing the *ex vivo* colon model experiment.
○ Thaw an aliquot of the FSI in the anaerobic chamber for a maximum of 15 min. It is important to minimise the thawing period to ensure that individual groups of bacteria, including *E. coli* do not have an opportunity to start growing.
○ Add 12 ml of the thawed FSI aliquot to 80 ml of double-strength Faecal Fermentation Medium (FFM) (Table 1) and mix gently, followed by dispensing an appropriate arbitrary volume (*e.g.* 10 ml) of the FSI/FFM mixture into universal sterilin tubes.

○ Ensure that the anaerobic chamber used for this whole procedure is maintained under strict anaerobic conditions prior to and during the preparation of the micro-Matrix cassette (image depicted in Fig. 2). Place an anaerobic indicator strip containing resazurin in the anaerobic chamber to confirm an anaerobic environment. Ensure that anaerobic gas mix (containing 10 % CO_2_ and 10 % H_2_ in nitrogen) and Technical-grade nitrogen cylinders supplying the anaerobic chamber, gloves and side ports have sufficient gas for all steps during preparation of the micro-Matrix cassette. Technical-grade nitrogen is of an adequate quality to create anaerobic conditions within the anaerobic chamber.

**Table 1:** **Recommended composition of Faecal Fermentation Medium (FFM) derived and adapted from Fooks and Gibson)**[9]. For micro-Matrix *ex vivo* colon model experiments, as an example, the preparation of 160 ml of FFM is advised for substrates whereby miniscule amounts of solid substrates are added directly to the FFM-FSI mix. As an example, the preparation of 80 ml of double-strength FFM is recommended for liquid substrates (or reconstituted solid substrates) whereby the liquid substrate is added in a 1:1 v/v ratio to the FFM-FSI mix. However, we advise users to calculate the volumes and concentrations of FFM that they require, depending on the specific needs and design of their experiments.

**Reagent**	**Concentration for FFM (mg/L) or (ml/L)**
Tryptone water	2000
Yeast extract	2000
Cysteine-HCl	1000
Bile salts	500
NaHCO_3_	2000
Tween 80	2 ml/L
Hemin	50 (dissolved in 3 drops of 1 M NaOH)
Vitamin K_1_	10 µl

**From autoclaved stock solutions**	

NaCl (10% w/v stock solution)	1 ml/L
KH_2_PO_4_ (4% w/v stock solution)	1 ml/L
K_2_HPO_4_ (4% w/v stock solution)	1 ml/L
CaCl_2_.6H_2_0 (4% w/v stock solution)	1 ml/L
MgSO_4_.7H_2_0 (1% w/v stock solution)	1 ml/L
○ Add an equal volume of the test substrates to the FSI/FFM mixture. For negative controls, add sterile water pre-equilibrated under anaerobic conditions to an equal volume of double-strength FFM.
○ Mix each of the samples from the step above by inverting 3–4 times, and then dispense 7 ml of each sample into designated bioreactor wells in the micro-Matrix cassette.
○ Prior to taking the T0 hour sample, gently mix the contents of each well by pipetting 1 ml volumes up and down three times each. This prevents precipitation of denser faecal material and/or other components to the bottom of the bioreactor wells and ensures that all aliquots are equal and contain a homogenous mix. Then take 2 × 1 ml aliquots for DNA extractions, metabolomics, proteomics and other such downstream applications. This ensures that 5 ml of sample remains in each bioreactor well at the start of the experiment.
○ After all T0 aliquots are taken, place the top plate with pre-assembled filter bars onto the micro-Matrix cassette with the metal clamps, ensuring that a perfectly hermetic seal is formed by visually inspecting to ensure that the top plate and clamps are perfectly straight and aligned. The lack of a perfectly hermetic seal can make the system amenable to contamination and can expose the individual bioreactors to environmental oxygen.
○ Once the micro-Matrix cassette-top plate unit is satisfactorily assembled, remove the assembled unit from the anaerobic chamber and assemble it onto the micro-Matrix unit using the clamps located adjacent to the orbital shaker. Assemble the gas bars onto the four rows carefully.
○ Commence the experiment using the online software with the three main parameter minimum and maximum settings as follows: i) Dissolved Oxygen (DO) set points between 0 and 150 % air saturation as the lower and upper limits respectively; ii) pH set points at pH 6.8 with lower and upper limits of pH 6.2 and pH 7.2 respectively and iii) temperature set points at 37 °C with lower and upper limits of 35 °C and 39 °C respectively. Conduct experiments with orbital shaking at 275 rpm to ensure that all sample mixtures remain in a homogenous mix throughout the duration of the experiment, thereby preventing precipitation of denser components and minimising the possibility of blocking the DO, pH and temperature sensors in each of the 24 bioreactors.
○ Switch on the liquid ammonia flow within one minute of commencing the experiment.
○ Monitor all real-time readouts of the three main parameters detected by the sensors (DO, pH and temperature), as well as dosage rates of BIP^R^ nitrogen, CO_2_ and liquid ammonia at regular intervals throughout the duration of the experiment.
○ Depending on the design of the experiment, pause the fermentation at designated time points (examples of which include T6hr, T24hr as arbitrary time points) to take aliquots, by repeating the steps described above for assembling the top plate and cassette unit.
○ At the end of the experiment, carefully remove the micro-Matrix cassette unit from the machine to take aliquots in the same manner as is described in the relevant steps above.
○ Perform the checklists on the instruction manuals for the micro-Matrix unit to save the fermentation parameter raw data readouts and switch off each of the gases including all associated valves for BIP^R^ nitrogen and liquid ammonia.

## Additional information

### Merits of the micro-Matrix bioreactor fermentation system for *ex vivo* colon model experiments

The micro-Matrix system is a small-scale high-throughput system containing 24 independent bioreactors (with a maximum capacity of 10 ml in each bioreactor well), enabling researchers to simultaneously assess 24 samples/substrates in a single experiment including appropriate controls. Images depicting the various components of the micro-Matrix system, including the bioreactor cassette/plate with built-in sensors for dissolved oxygen (DO), temperature and pH are shown in Fig. 2. The micro-Matrix bioreactor fermentation system enables researchers to control DO, pH and temperature to precise set points. It can potentially be used as an *ex vivo* model of the human distal colon and the fermentation parameters can be pre-set to mimic *in vivo* conditions. Strict anaerobic conditions are created by forming a hermetic seal using the micro-Matrix bioreactor cassette/plate, top plate and filter bars assembled, as well as the addition of nitrogen to ensure that the dissolved oxygen concentrations remain in the range of 0–150 % air saturation (preferably within the lower range as close as possible to 0 % air saturation, mimicking strict anaerobic conditions). The pH settings are controlled to stay within the lower and upper ranges between pH 6.2 to pH 7.2 in order to ensure that the pH values remain as close as possible to pH 6.8 in each of the 24 independent bioreactors, to resemble physiological conditions found in the human distal colon. The pH values are controlled by the addition of either CO_2_ or 20 % v/v liquid ammonia to decrease or increase the pH respectively. Finally, the temperature is set at 37 °C throughout the experiments to mimic conditions in the human distal colon.

Overall, the micro-Matrix bioreactor fermentation system for conducting *ex vivo* colon model experiments offers researchers numerous advantages. Most notably, the primary advantage is the high-throughput nature of the system, enabling researchers to evaluate 24 independent substrates including controls in each experiment. This is in contrast to other systems which usually only enable researchers to assess 3–4 samples in *ex vivo* colon model experiments [Table 2]. Therefore, additional technical replicates can be incorporated into experiments using the micro-Matrix bioreactor fermentation system, which bolsters statistical analyses when compared to lower-throughput bioreactor systems. Another key advantage is the small scale nature of the micro-Matrix bioreactor fermentation system, with a maximum capacity of 10 ml in each bioreactor. This can be beneficial whereby limited amounts of substrates are available for assessment, such as purified peptides, antimicrobials, drugs and any other substrates which may be laborious and costly to purify. Thus, in contrast to the bench-top fermentation systems, *ex vivo* colon model experiments using the micro-Matrix bioreactor fermentation system can be significantly scaled down. Scaling down of such experiments should in principle yield the same outputs and results as medium to large scale fermentation systems, whilst offering the advantage of being higher-throughout and less laborious than other systems. Moreover, the scaled down aspect of the micro-Matrix system enables researchers to collect smaller quantities of faecal samples from volunteers, which may or may not be pooled, prior to performing such experiments. Oftentimes, medium and large scale fermentation systems require larger quantities of faecal samples to be collected from participants, along with the preparation of larger quantities (in the magnitude of Litres) of faecal fermentation media (FFM). Amongst other advantages of the micro-Matrix bioreactor fermentation system include its technologically modern user-friendly software system enabling automated and precise control of the parameters during fermentation experiments, precluding the need for laborious manual attachment and assembly of larger more complicated fermentation systems and vessels. Finally, the single-use and disposable nature of the micro-Matrix bioreactor cassette precludes the need for laborious autoclaving and cleaning of larger fermentation vessels, prior to the next experiment.

**Table 2:** Comparison of the different features of six bioreactor systems used to model the colon.

Features	micro-Matrix	MiCoMo	MiGut	RoboGut	TIM-2	SHIME
*Throughput*	High (24 tests per experiment)	Medium (3 tests per experiment)	Medium (4 tests per experiment but scalable)	Medium (4 tests per experiment)	High (10 tests per experiment)	Low (1 test per experiment)
*Fermentation type*	Batch	Batch Fed	Continuous	Continuous	Continuous/semi-continuous	Semi-Continuous
*Locations of GIT modelled*	One; distal colon	One; colon	Three; ascending, transverse and descending colon	One; distal colon	Proximal (ascending) colon primarily)	Five; stomach, small intestine, ascending, transverse and descending colon
*Faecal donor(s)*	Pooled donors	Individual	Pooled or individual donors	Individual	Individual	Individual
*Microbiota adaptation time*	No	Yes; 3–5 days	Yes; 10–14 days	Yes; 1–3 days as batch culture, followed by 14–28 days continuous culture	Yes; typically 16 h	Yes; typically 14 days
*Duration of experiment post adaptation*	Typically 24–48 h	Typically 2 weeks	Option up to 7 weeks	Typically weeks	Typically 1 week	Typically weeks
*Set* pH *(to represent* pH *conditions in the colon location being modelled)*	pH 6.8	pH 6.7	Ascending colon pH 5.4–5.6, transverse colon pH 6.15–6.36, descending colon pH 6.65–6.85	Neutral, to reflect pH in colon	pH 5.8 in proximal (ascending) colon	Ascending colon pH 5.6–5.9, transverse colon pH 6.1–6.4, descending colon pH 6.6–6.9
*Reagents used for* pH *control*	20 % v/v liquid NH_3_ (alternatively NaOH) CO_2_	NaOH HCl	NaOH HCl	5 % (v/v) NaOH 5 % (v/v) HCl	1 M NaOH HCl	1 M NaOH
*Gases used to maintain anaerobic conditions*	BIP-grade N_2_	N_2_	N_2_	N_2_	N_2_	90 % N_2_, 10 % CO_2_
*Dialysate system*	No; but can include separate SGID including dialysis	No	No	No	Yes	No
*Mucosal component*	No	Optional	Yes	No	No	Yes, optional
*Source of faecal material*	Any cohort	Any cohort	Any cohort	Any cohort	Any cohort	Any cohort
*Examples of References reporting the system*	Strain et al. 2023 [10]	Jin et al. 2022 [11]	Davis Birch et al. 2023 [12]	Gianetto-Hill et al. 2023 [8]	Venema 2015 [13]	Van de Wiele et al. 2015 [14]

Although the aforementioned advantages associated with the micro-Matrix bioreactor fermentation system are attractive to researchers wishing to conduct *ex vivo* colon model experiments, issues relating to *E. coli* blooms are often encountered with the system. Here we specifically focus on and outline some of the key factors that contribute to *E. coli* blooms in *ex vivo* colon model experiments conducted using the micro-Matrix bioreactor fermentation system and describe some mitigating approaches.

## Factors contributing to *E. coli* blooms in *ex vivo* colon model experiments, specifically involving the use of the micro-Matrix bioreactor fermentation system, and mitigating options thereof

### Faecal sample collection to prepare frozen standardised inoculum (FSI)

This is a critical first step in the whole process of conducting *ex vivo* colon model experiments. Typically, 6–10 volunteers are enrolled to donate faecal samples and for the majority of studies, volunteers who are in good health, have not taken any antibiotics, proton pump inhibitors (PPIs) and/or any other medication that may affect the composition of their gut microbiomes, over the last 6 months. In many jurisdictions and for publications in high impact peer-reviewed journals, it is necessary to obtain ethical approval for collecting faecal samples. We recommend users to include an option to collect faeces on more than one occasion from each of the volunteers for the project/study. Having the option to collect faecal samples on more than one occasion facilitates the preparation of additional, albeit not identical, frozen standardised inoculum (FSI) preparations. This helps to offset the risk of *E. coli* blooms following prolonged storage of FSI aliquots at −80 °C for durations longer than approximately 6 months or in the event that all FSI is used during early stages of the project.

With regards to the composition of the baseline gut microbiome from different FSI batches, we recommend users to test identical substrates/samples using multiple batches of FSI (derived from faecal samples obtained from a different set of volunteers). This approach will provide biological replicates for statistical analyses. It will also help to confirm whether the alterations caused to the gut microbial community as a consequence of the test substrates are consistent across different FSI preparations, irrespective of the initial microbial composition of the starting gut microbiome profile and which volunteers donated the faecal samples.

**Fig. 3:**
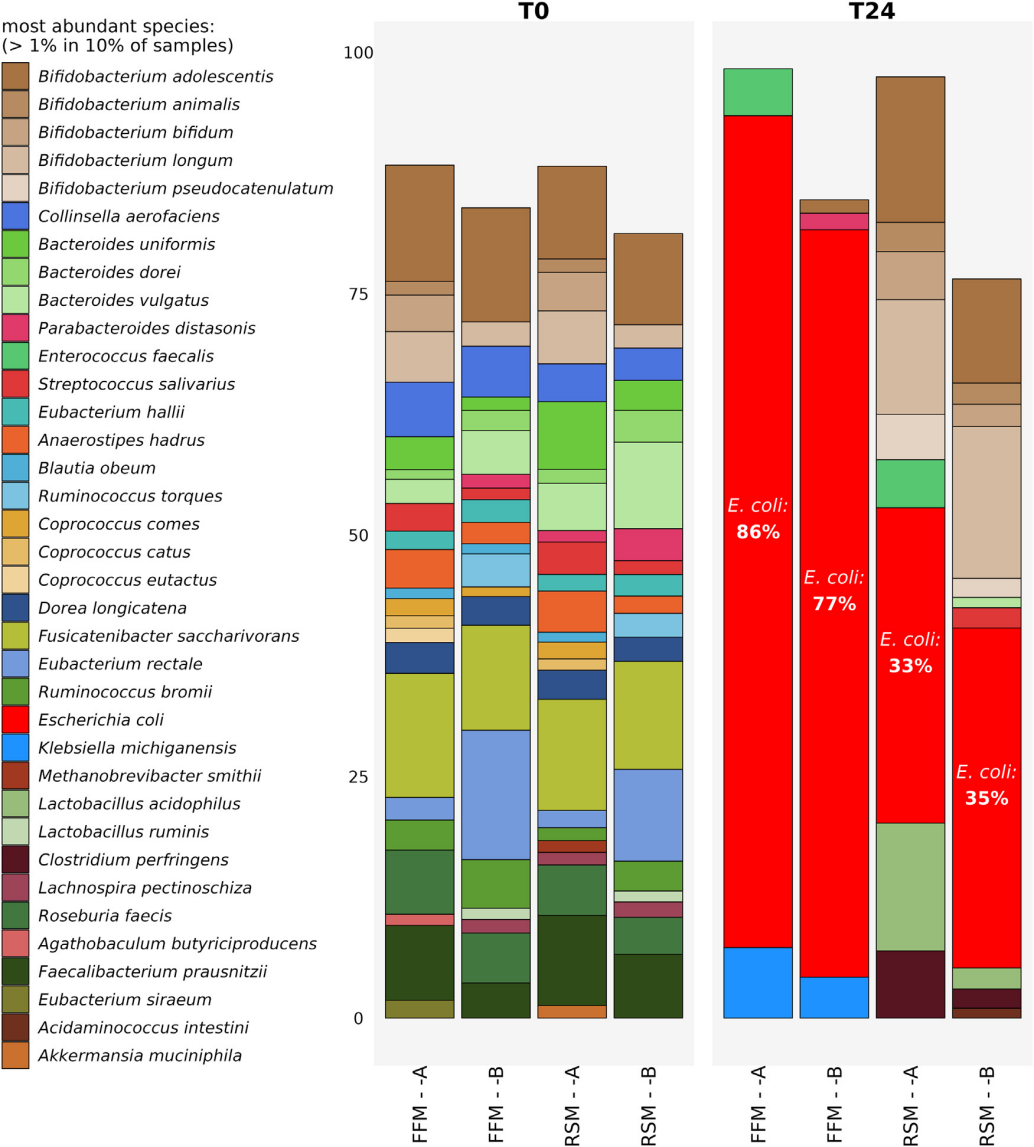
Figure demonstrating the occurrence of *E. coli* blooms from two different batches of FSI labelled ‘A’ and ‘B’ derived from two different sets of donors. Species relative abundance as determined through shotgun metagenomics analysis using MetaPhlAn3. FFM denotes faecal fermentation medium and RSM denotes a final concentration of 5% w/v Reconstituted Skim Milk. T0 and T24 denote samples taken at the start and at the end of the experiment respectively. *E. coli* is depicted in red and % relative abundance of *E. coli* is shown for T24. The most abundant species with a relative abundance of >1 % in 10 % of samples are shown. Raw data for this Figure has been deposited in the European Nucleotide Archive (ENA) at EMBL-EBI under accession number PRJEB64001 (https://www.ebi.ac.uk/ena/browser/view/PRJEB64001).

In an effort to identify the root cause of *E. coli* blooms encountered in such studies, we performed a proof of concept experiment whereby we tested identical substrates in the same micro-Matrix *ex vivo* colon model experiment, using two different batches of FSI (with faecal samples derived from volunteers recruited as part of two different studies, within the remits of the approved ethics applications for studies APC108 and APC097). These two batches of FSI were prepared using identical SOPs by two different researchers within approximately two weeks of each other, and the micro-Matrix *ex vivo* colon model experiment was conducted approximately 14 months after preparing the FSI batches. Fig. 3 shows that irrespective of the subtle differences in the composition of the starting T0 microbiome profiles in the two different batches of FSI, *E. coli* blooms still occurred at T24 with both FSI preparations when testing identical substrates in the same micro-Matrix *ex vivo* colon model experiment. This indicates that *E. coli* blooms occurred when two independent FSI sources, which we made using the same protocol and stored for approximately 14 months at −80 °C were used which implies that some complex interrelated aspects of the *ex vivo* colon model protocol are responsible for the blooms and that it is not related to the actual source of the faecal material.

### Effects of prolonged storage of FSI at −80 °C

**Fig. 4:**
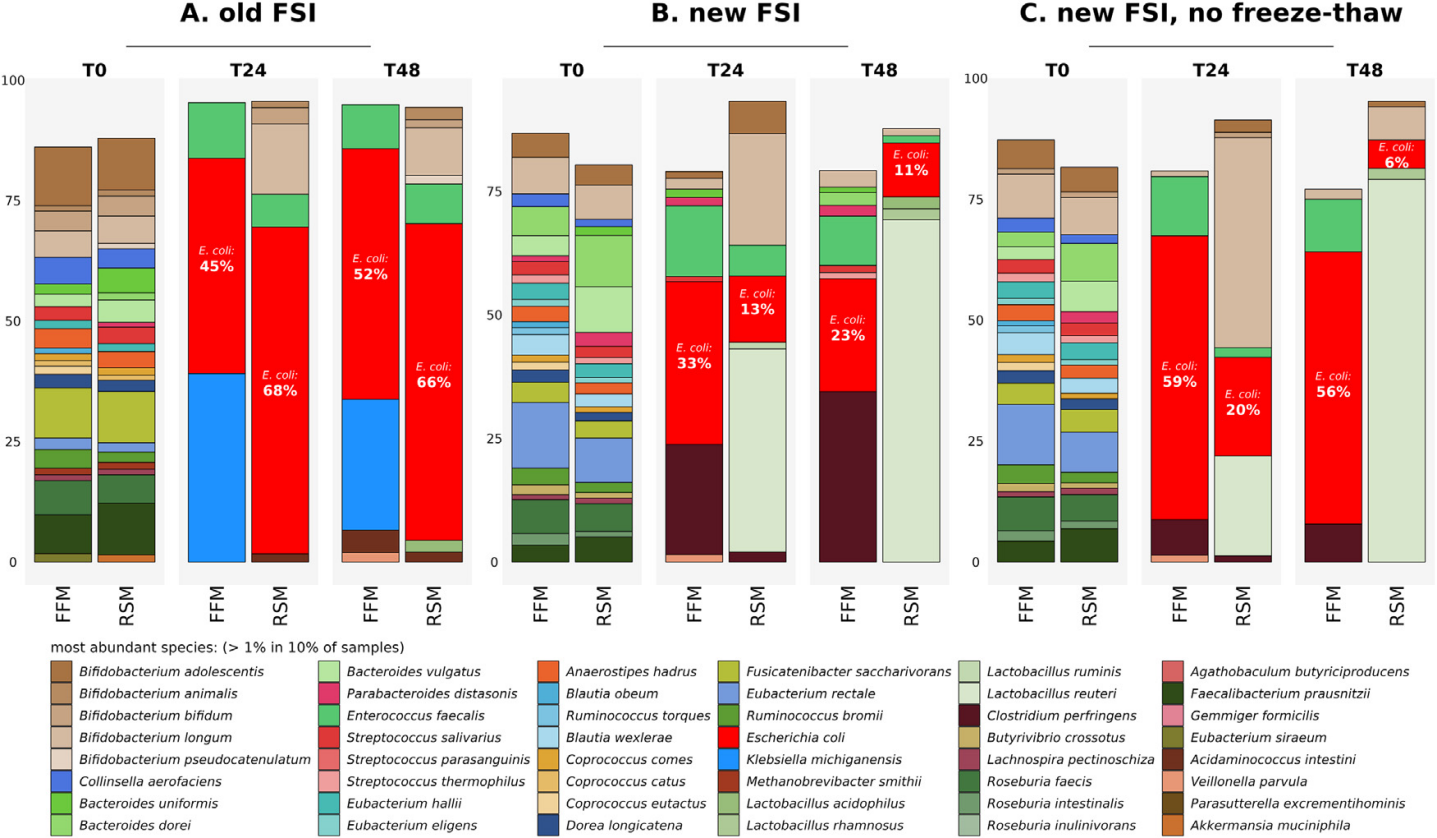
Figure demonstrating the occurrence of *E. coli* blooms from A) a batch of FSI stored at −80 °C for a duration longer than 6 months; B) from a batch of FSI stored at −80 °C for less than 2 months prior to the micro-Matrix experiment and C) from a batch of ‘freshly prepared FSI’ which was incubated in an anaerobic chamber at 37 °C for an additional two hours without being subjected to a freeze/thaw cycle. Species relative abundance as determined through shotgun metagenomics analysis using MetaPhlAn3. FFM denotes faecal fermentation medium and RSM denotes a final concentration of 5% w/v Reconstituted Skim Milk. T0, T24 and T48 denote samples taken at the start, in the middle (24 hrs) and at the end (48 hrs) of the experiment respectively. *E. coli* is depicted in red and % relative abundance of *E. coli* is shown for T24 and T48. The most abundant species with a relative abundance of >1 % in 10 % of samples are shown. Raw data for this Figure has been deposited in the European Nucleotide Archive (ENA) at EMBL-EBI under accession number PRJEB64001 (https://www.ebi.ac.uk/ena/browser/view/PRJEB64001).

O'Donnell and co-workers recommend that the FSI aliquots are stored and used for *ex vivo* colon model experiments within 8 weeks of preparation [1,2]. Therefore, if practical, we concur with this recommendation that the FSI aliquots are used within this time frame. *E. coli* blooms are more likely to occur after extended storage of the FSI at −80 °C. Fig. 4A highlights an example of an *E. coli* bloom issue (as well as a bloom of other coliforms such as *Klebsiella michiganensis*) we encountered when FSI aliquots stored at −80 °C for longer than 6 months were used in the *ex vivo* colon model experiments. In contrast, Fig. 4B highlights the attenuation of *E. coli* blooms in *ex vivo* colon model experiments where FSI was used within approximately two months of preparation. This indicates that use of FSI within 6–8 weeks of preparation is likely to significantly reduce the probability of encountering *E. coli* blooms, as is recommended by O'Donnell and co-workers [1,2].

### Freezing and thawing of the FSI prior to commencing the micro-Matrix *ex vivo* colon model experiment

We performed a proof of concept experiment where a ‘fresh’ unfrozen aliquot of the FSI, prepared on the day that faecal samples were donated, was held in the anaerobic chamber for two hours at 37 °C on the day of preparation, prior to commencing the micro-Matrix experiment. This resulted in a somewhat higher relative abundance of *E. coli* (Fig. 4C) compared to experiments subsequently conducted with frozen aliquots of the same batch of FSI (example previously shown in Fig. 4B described above) which were thawed in an anaerobic chamber for 10 min prior to adding them to the faecal fermentation medium (Fig. 4B). Our hypothesis is that incubation of the FSI for two hours at 37 °C in an anaerobic chamber without freezing may have provided a selective advantage to *E. coli* allowing cells to acclimatise to the conditions pertaining in the faeces following excretion, thereby growing rapidly in the subsequent fermentation to dominate the microbiota (Fig. 4C).

### *E. coli* blooms occur within the first six hours in micro-Matrix *ex vivo* colon model experiments

**Fig. 5:**
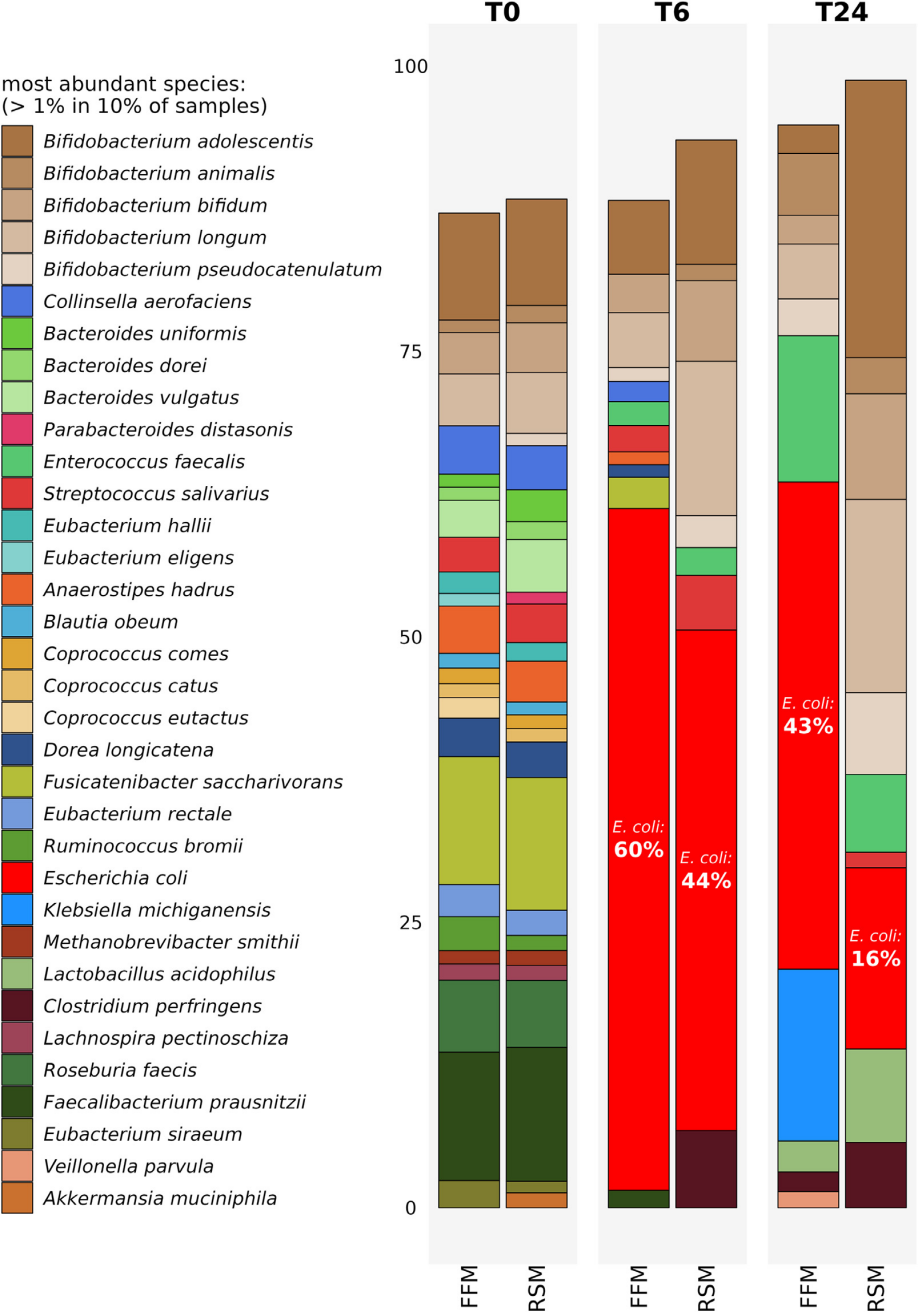
Figure demonstrating the occurrence of *E. coli* blooms within 6 hrs of commencing the faecal fermentation experiment. Species relative abundance as determined through shotgun metagenomics analysis using MetaPhlAn3. FFM denotes faecal fermentation medium and RSM denotes a final concentration of 5% w/v Reconstituted Skim Milk. T0, T6 and T48 denote samples taken at the start, 6 hrs after commencing the fermentation and at the end (48 hrs) of the experiment respectively. *E. coli* is depicted in red and % relative abundance of *E. coli* is shown for T24 and T48. The most abundant species with a relative abundance of >1 % in 10 % of samples are shown. Raw data for this Figure has been deposited in the European Nucleotide Archive (ENA) at EMBL-EBI under accession number PRJEB64001 (https://www.ebi.ac.uk/ena/browser/view/PRJEB64001).

Although *E. coli* blooms are often apparent at T24 of *ex vivo* colon model experiments in terms of relative abundance, our sequencing experiments demonstrated that such blooms were present in samples taken at the T6 time point after commencement of the experiment as well (Fig. 5). Therefore, it is apparent that conditions favouring the growth of *E. coli* over other genera and species represented in the gut microbiome, occur relatively early in such experiments.

### Dissolved oxygen (DO) concentration as a factor contributing to *E. coli* blooms

The level of DO is another important factor which can potentially contribute to *E. coli* blooms when using the micro-Matrix bioreactor fermentation system. Although the pre-programmed settings in the micro-Matrix system incorporates nitrogen throughout the experiment to maintain the DO concentration as low as possible and to compensate for potentially high starting levels of DO, the time lag involved in decreasing the DO levels to within the optimum ranges is likely to give facultative anaerobes such as *E. coli* a competitive advantage over strict obligate anaerobes such as *Bifidobacterium* species, likely facilitating *E. coli* outgrowth. In an effort to control DO, all substrates, reagents and materials should be stored under anaerobic conditions for between 2 and 18 hrs, prior to commencing the experiment. This should help ensure that the starting DO concentrations are as low as possible to mimic strict anaerobic conditions found in the human distal colon. Examples of items which can be stored overnight under anaerobic conditions prior to commencing the *ex vivo* colon model experiment include all plastic-ware, as well as the FFM that is used as a basal medium. Test substrates can also be stored under anaerobic conditions for between 2 and 18 hrs, depending on the specific nature of the substrates and whether they contain live microbes or not. If the substrates contain live microbes and/or if they are amenable to microbial contamination, we recommend storing them under anaerobic conditions inside the anaerobic chamber for approximately 2 hrs prior to commencing the experiment.

### Incorporation of static INFOGEST/Simulated Gastrointestinal Digestion (SGID) protocols in *ex vivo* colon model experiments

Following consumption, food is subjected to digestion as it passes through the gastrointestinal tract. This results in the release of small molecules from the food, such as simple sugars, amino acids, fatty acids and their absorption into the blood stream. Therefore, to mimic *in vivo* situations, we recommend users to subject their test substrates to a Simulated Gastrointestinal Digestion (SGID) protocol, such as the globally-harmonised INFOGEST protocol [3,4] to digest the food material, followed by dialysis to mimic absorption of small molecules. We also hypothesise that the presence of simple sugars including glucose, galactose and lactose and amino acids in substrates being tested in *ex vivo* colon model experiments is likely to provide a selective growth advantage to *E. coli,* thus facilitating the development of blooms. However, inclusion of the dialysis step should mitigate this by reducing the concentration of such molecules in the *ex vivo* colon model.

Depending on the specific nature of the substrate, such *E. coli* blooms can potentially be attenuated by decreasing the concentrations of simple sugars such as glucose (180 Da), free amino acids and low molecular weight compounds by subjecting the samples to dialysis across 500 Da membranes for a duration of 24–30 hrs. However, dialysis may also inadvertently remove or reduce the concentrations of other key low molecular weight metabolites weighing less than 500 Da and thus, affect the data obtained. Therefore, there is a fine balancing act between removing/reducing the concentrations of small molecules and maintaining key metabolites which typically reach the colon in *in vivo* situations. Following dialysis, we also recommend lyophilising the dialysed samples and reconstituting the lyophilised samples in the original starting volume of the substrate in sterile distilled water.

### Food substrates subjected to static INFOGEST/Simulated Gastrointestinal Digestion (SGID) protocols

**Fig. 6:**
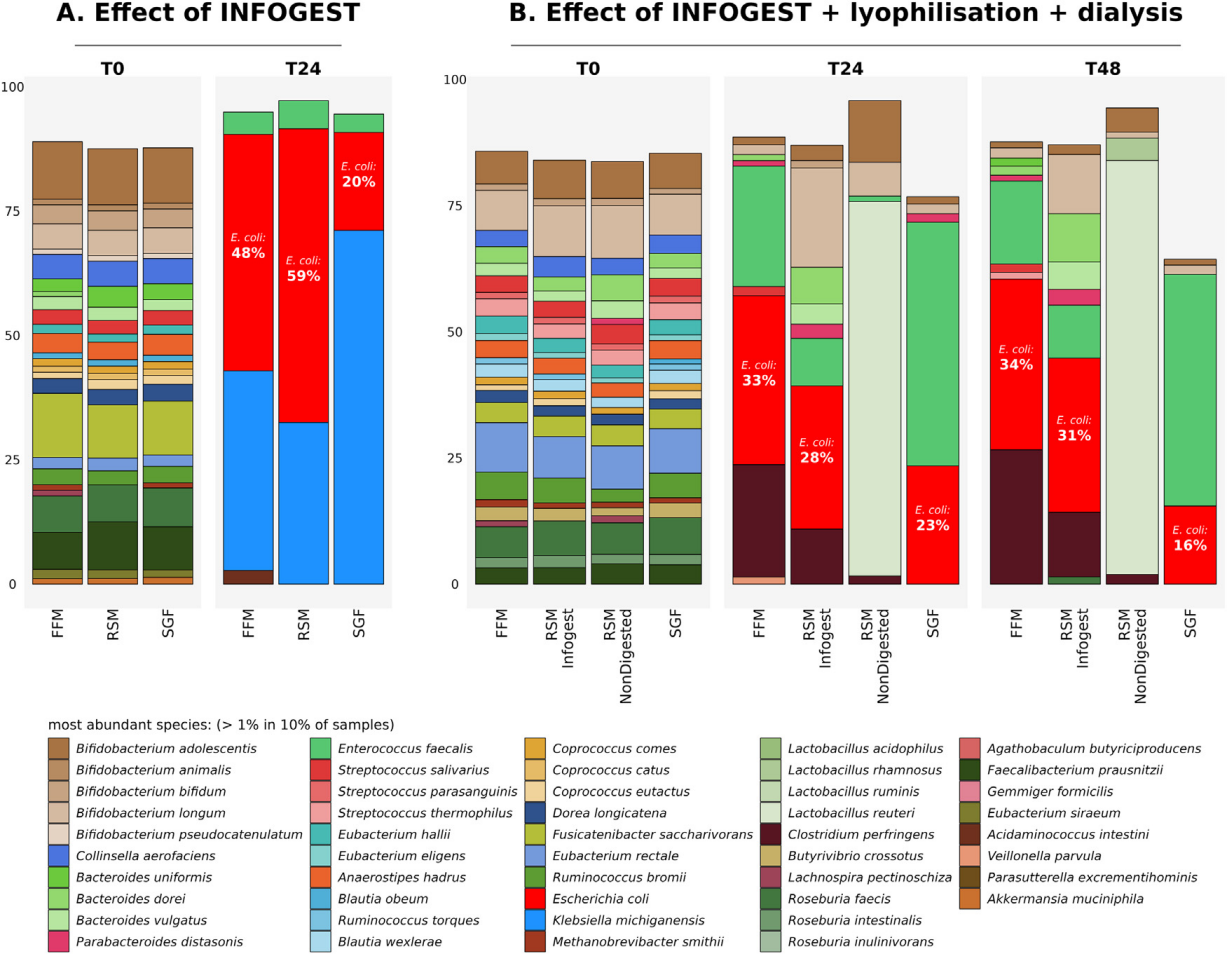
Figure demonstrating the occurrence of *E. coli, Klebsiella michiganensis* and *Enterococcus faecalis* blooms with A) samples subjected to an SGID (INFOGEST) protocol and B) the attenuation of *E. coli* blooms as a consequence of optimisation of the post-INFOGEST processing of samples. Species relative abundance as determined through shotgun metagenomics analysis using MetaPhlAn3. FFM denotes faecal fermentation medium and RSM denotes a final concentration of 5% w/v Reconstituted Skim Milk. SGF denotes Simulated Gastric Fluid, Simulated Intestinal Fluid and the basal salts and enzymes used for all INFOGEST procedures. T0, T24 and T48 denote samples taken at the start, at the middle (24 hrs) and at the end (48 hrs) of the experiment in Fig. 6A and B. *E. coli* is depicted in red and % relative abundance of *E. coli* is shown for T24 and T48.The most abundant species with a relative abundance of >1 % in 10 % of samples are shown. Raw data for this Figure has been deposited in the European Nucleotide Archive (ENA) at EMBL-EBI under accession number PRJEB64001 (https://www.ebi.ac.uk/ena/browser/view/PRJEB64001).

We recommend that the INFOGEST protocol is applied to digest samples as described by Brodkorb et al. [3] followed by dialysis for 24–30 h using four water changes at regular intervals. It is advised that the digested substrate inside the dialysis tubing is immersed in a volume approximately 100 times the volume of the digested substrate *e.g.* a 30 ml starting volume of a digested substrate should be immersed in approximately 3 L of distilled water to facilitate efficient dialysis across the membrane. Since dialysis results in osmosis across the membrane, the volume inside the dialysis tubing increases, and therefore the volume of distilled water that the tubing is immersed in must also be adjusted to compensate for this increased volume, with a view to maintaining an efficient gradient across the membrane at all times. Overall, a 24–30hr dialysis procedure results in an estimated 10-fold dilution of the original substrate volume. Therefore, we recommend lyophilising the digested dialysed samples to remove the excess water, and reconstituting the lyophilised powders in the original starting volume with sterile distilled water (to counteract the effects of dilution due to osmosis) prior to testing them in micro-Matrix *ex vivo* colon model experiments. Fig. 6A shows an example of the common *E. coli* bloom phenomenon when testing INFOGEST digested substrates, without optimising the dialysis and lyophilisation procedures. The presence of *E. coli* blooms as well as growth of *Klebsiella michiganensis* and *Enterococcus faecalis* at T24 is apparent in Fig. 6A. In contrast, Fig. 6B shows an example of a partial attenuation of *E. coli* blooms after optimising the dialysis and lyophilisation procedure post-INFOGEST.

## Use of viable plate counts as a proxy for predicting relative abundance microbiome results

Although the genera associated with *ex vivo* colon model experiments vary widely, the three genera typically most dominant in terms of relative abundance microbiome results observed after 24hr or 48hr in our *ex vivo* colon model experiments were *Escherichia, Lactobacillus* and *Bifidobacterium.* Viable plate counts for these genera may be a useful approach to obtaining timely data as to the abundance of each of these three groups of bacteria in the microbiome post application of the *ex vivo* colon model, while being cognisant of the advantages and limitations of this methodology. Therefore, we recommend using both metagenomics and culturomics approaches in concert, with the use of viable plate count data as a proxy to facilitate early identification of *E. coli* blooms and consequent high relative abundance of *E. coli* in downstream shotgun sequencing data. The use of viable plate counts is an inexpensive method to predict *E. coli* blooms and may assist researchers in making decisions about whether to sequence the samples or not, especially if there are budget constrains in the study/project. We recommend using Violet Red Bile Agar (VRBA) for enumerating total coliforms cfu/ml counts, Difco LBS agar to enumerate total *Lactobacillus* cfu/ml counts and *Bifidobacterium* Specific Medium (BSM) and its BSM supplement to enumerate total *Bifidobacterium* cfu/ml counts from faecal sample aliquots at various time points throughout the *ex vivo* colon model experiment. We recommend incubating VRBA plates at 37 °C for 24 hrs under aerobic conditions, whereas LBS agar and BSM agar should be incubated at 37 °C for 48 hrs under anaerobic conditions for total *Lactobacillus* and total *Bifidobacterium* cfu/ml counts respectively.

Depending on the specific nature and objectives of the research project, we also recommend taking samples at the start (T0hr), middle (T24hr) and end (T48hr) of *ex vivo* colon model experiments and subjecting them to quantitative PCR (qPCR) using either general universal bacterial 16S primers or species-specific primers, in lieu of or as an adjunct to shotgun metagenomics sequencing approaches.

A summary of the key factors contributing to *E. coli* blooms in micro-Matrix experiments is included in Table 3.

**Table 3:** Summary of factors contributing to *E. coli* blooms and methods to mitigate such blooms.

Factor contributing to *E. coli* blooms	Recommended methods to mitigate *E. coli* blooms
Prolonged storage of FSI at −80 °C	Obtain ethical approval for collecting faecal samples from volunteers on multiple occasions during the course of the project/study.
	Perform all micro-Matrix experiments within 8 weeks of preparing aliquots of FSI if possible and practical.
	Perform Quality Control (QC) checks at regular intervals to assess microbial biodiversity in FSI aliquots.
Prolonged thawing of FSI	Thaw for minimum amount of time as possible (10–15 min) in anaerobic chamber.
Presence of high concentrations of simple low molecular weight sugars/free amino acids following INFOGEST	Perform dialysis for 24–30 hrs with regular replacement of water, using 500 Da dialysis membranes post-INFOGEST.
	After dialysis, perform lyophilisation to negate dilution of digested substrates post-INFOGEST.
	After lyophilisation, reconstitute dialysed digested substrates in original starting volume with water to negate any dilution.
High starting DO concentrations in substrates/FFM mix	Pre-equilibrate FFM, water, reagents, universal sterilins, falcon tubes, pipette tips and all other plastic-ware overnight under anaerobic conditions before commencing experiments.
High starting levels of *E. coli* in FSI	Perform QC checks from T0 samples and/or sequence aliquots of FSI at regular intervals to assess microbial biodiversity and *E. coli* relative abundance.
	Recruit volunteers who are in good health and have not taken antibiotics, proton pump inhibitors and/or other medication possibly leading to loss of microbial diversity/loss of strict anaerobes/dysbiosis, as a confounding factor.
	Perform regular QC checks by using FSI aliquots from other projects within the department (within the remit of ethical approval documentation) for sequencing QC checks and detection of *E. coli* blooms.
pH either too high or too low	Maintain pH equilibrium within the 6.2–7.2 range by using 20 % v/v liquid ammonia and CO_2_ to increase and decrease pH respectively, throughout the experiment.
Preparation of FFM	Prepare FFM as close as possible to the day of the micro-Matrix experiment.
	Store at 4 °C for a maximum of 7 days prior to commencing the micro-Matrix experiment.

## Conclusions

In conclusion, there is a complex interplay of factors that contribute to *E. coli* blooms in *ex vivo* colon model experiments, and this is expected to be even more apparent in a small-scale highly sensitive platform such as the micro-Matrix bioreactor fermentation system. By far, one of the most crucial factors investigated is the storage of FSI preparations at −80 °C for periods longer than 6 months. This is followed by other factors such as the presence of simple sugars in test substrates, high DO values, aberrant pH values and prolonged thawing of FSI aliquots. Thus, if possible and practical, such investigative *ex vivo* colon model experiments should be completed within 8 weeks of preparing FSI batches, as is advocated by O'Donnell and co-workers [1,2]. The presence of simple sugars and/or free amino acids as an important factor contributing to *E. coli* blooms can be mitigated by incorporating appropriate steps such as dialysis and lyophilisation of substrates, either with or without subjecting these substrates to SGID protocols. Another crucial factor which may contribute to *E. coli* blooms is the lack of anaerobic conditions before and during the course of the *ex vivo* colon model experiment. This factor can be minimised by pre-equilibrating all reagents, substrates and plastic-ware under strict anaerobic conditions before commencing the experiment to ensure that the starting DO concentrations at the beginning of the experiment are as low as possible.

In addition to the above-mentioned mitigation strategies, we also recommend the use of stringent positive and negative controls in every micro-Matrix *ex vivo* colon model experiment, which can serve as reliable early indicators of *E. coli* blooms and can provide insights into the most likely factors at play, should such *E. coli* blooms occur. Examples of negative controls include the basal media FFM/FSI mixture (either without any substrate or only with sterile water as a substrate) in every *ex vivo* colon model experiment to identify the FFM/FSI mixture as a potential source or factor causing *E. coli* blooms. Examples of positive controls can include known probiotic mixtures containing a specific strain/mixture of strains or a mock community to monitor their growth throughout the *ex vivo* colon model experiment (and which can subsequently be verified by sequencing approaches to track their growth). Other examples of positive controls may include prebiotics such as inulin, fructo-oligosaccharides (FOS) or galacto-oligosaccharides (GOS), which are known to have a bifidogenic effect [5,6,7]. By including such positive controls, the growth of bifidobacteria can be tracked throughout the experiment, as a test of assay functionality and accuracy of the micro-Matrix bioreactor fermentation system as a true *ex vivo* model of the human distal colon and reliable predictor of what is likely to occur in *in vivo* situations. Finally, examples of other controls which could be incorporated to assess *E. coli* booms include antibiotics or antimicrobials such as colicins which prevent the growth of *E. coli*, in order to test assay functionality and perform QC checks.

If early predictive signs of *E. coli* blooms persist in such *ex vivo* colon model experiments, detected by inexpensive methods such as viable plate counts and/or other techniques, we recommend that users sequence a relatively small subset of extracted DNA samples to confirm these sightings and to understand how other members of the gut microbiome are responding. Overall, although the micro-Matrix bioreactor fermentation system is not exempt from being amenable to *E. coli* blooms, even where the mitigation strategies investigated here are applied, the system also has numerous advantages especially on account of its high-throughput nature, which is highly advantageous for large-scale screening projects. However, where extensive blooms are observed, we recommend that the experiments be repeated as it is not possible to draw meaningful conclusions where one or a small number of bacterial species are dominating the microbiome. Indeed, an unexpected bloom of any genus including *Escherichia* or *Lactobacillus* in micro-Matrix experiments is problematic and may indicate that the outputs of the particular experiment are not reflective or predictive of physiological situations.

The key focus of the method reported here is that it is high-throughput with a short experimental duration; currently 24 samples per experiment each with a duration of 48 h. Two key differences between this and previously reported models (Table 2) is that it does not include a microbiota adaptation time and is a batch fermentation system. Other models use an adaptation time ranging from 16 h to a number of weeks. This adaptation time enables the microbial community present in the faeces to re-establish itself in the laboratory bioreactor. This step was incorporated into the model systems as it was noted that fast growing species of bacteria, such as *E. coli* tend to dominate the population in the early phase of the allocated adaptation time. In addition, the existing models are all either batch-fed or continuous fermentation systems. Batch/continuous feeding was incorporated to more realistically mimic the *in vivo* physiological situation in the human colon where new material enters on a regular basis and also to provide nutrients on a continuous basis to support the growth and stabilisation of the microbiome within the bioreactor. Future developments of the micro-Matrix system will have to consider how to incorporate an adaptation time and batch/continuous feed and it is anticipated that such changes could significantly improve the model and lead to significant reduction in *E. coli* or other bacterial blooms. In addition, future projects using the micro-Matrix system may involve testing a variety of other FFM compositions reported in the literature, a very useful summary of which is provided in a Supplementary Table by Gianetto-Hill et al. 2023 [8] to determine whether alternative FFM compositions are more appropriate or not at mitigating *E. coli* blooms in micro-Matrix experiments.

## Ethics statements

Informed consent was obtained from subjects who donated faecal samples for this study. This work was approved by the Clinical Research Ethics Committee (project numbers APC108 and APC097).

## CRediT authorship contribution statement

**Harsh Mathur:** Conceptualization, Methodology, Writing – original draft. **Monica A. Mechoud:** Methodology, Writing – review & editing. **Chloe Matthews:** Data curation, Visualization, Writing – review & editing. **Cathy Lordan:** Data curation, Visualization, Writing – review & editing. **Jamie A. FitzGerald:** Data curation, Visualization, Writing – review & editing. **Tom Beresford:** Conceptualization, Supervision, Writing – review & editing. **Paul D. Cotter:** Conceptualization, Supervision, Writing – review & editing.

## Declaration of Competing Interest

The authors declare that they have no known competing financial interests or personal relationships that could have appeared to influence the work reported in this paper.

## Data Availability

The raw sequencing data has been submitted to ENA and the hyperlink is provided in the manuscript.

## Appendix Supplementary materials

Application, image 1

Application, image 2

Application, image 3

Application, image 4

Image, application 5
