# Supplementary figures and images for: Methods to mitigate Escherichia coli blooms in human ex vivo colon model experiments using the high throughput micro-Matrix bioreactor fermentation system
Source: MethodsX. 2023 Oct 6;11:102393. doi: 10.1016/j.mex.2023.102393 (PMC10577065; doi:10.1016/j.mex.2023.102393)

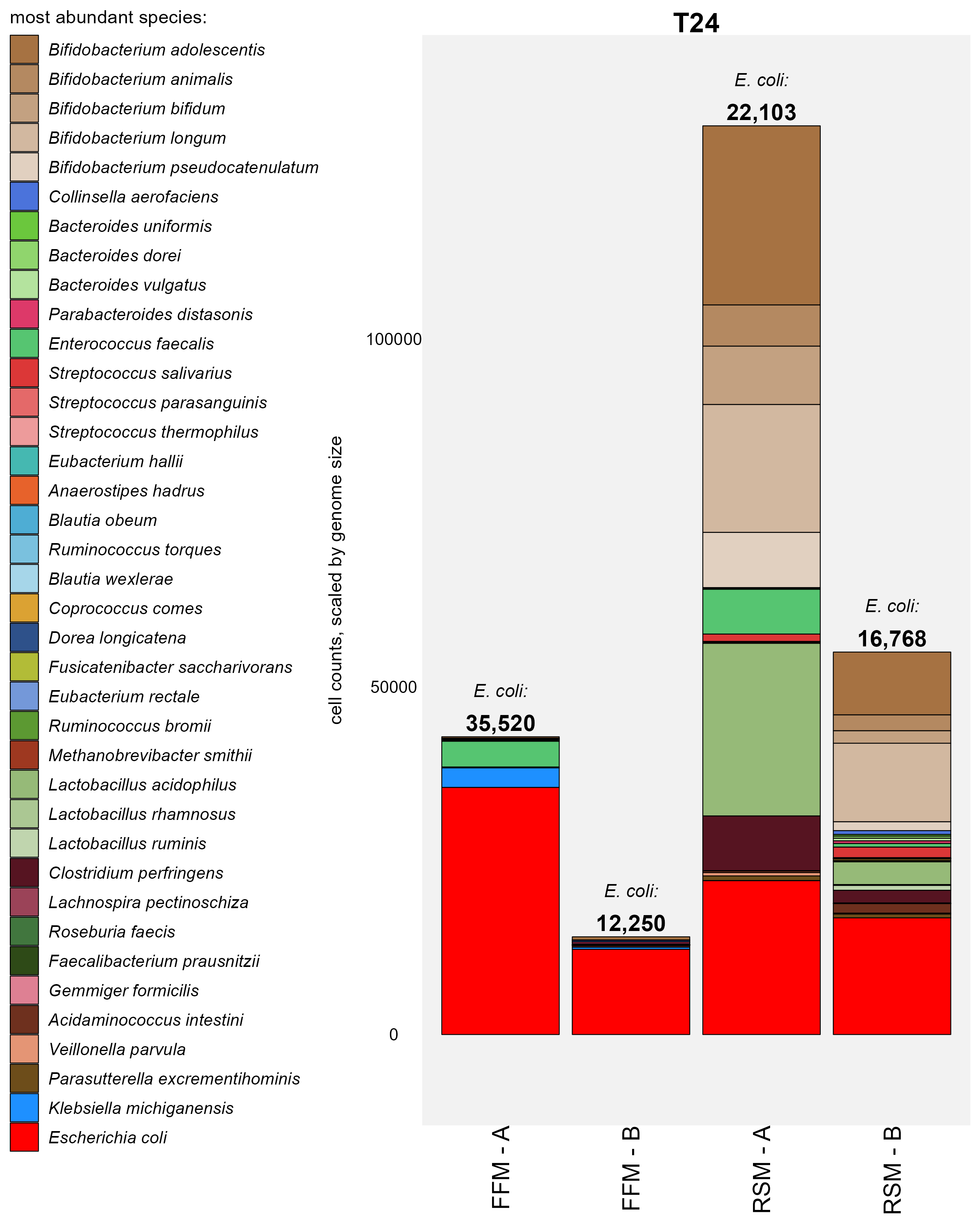

Supplement: Supplementary file 1 [file mmc1.docx]

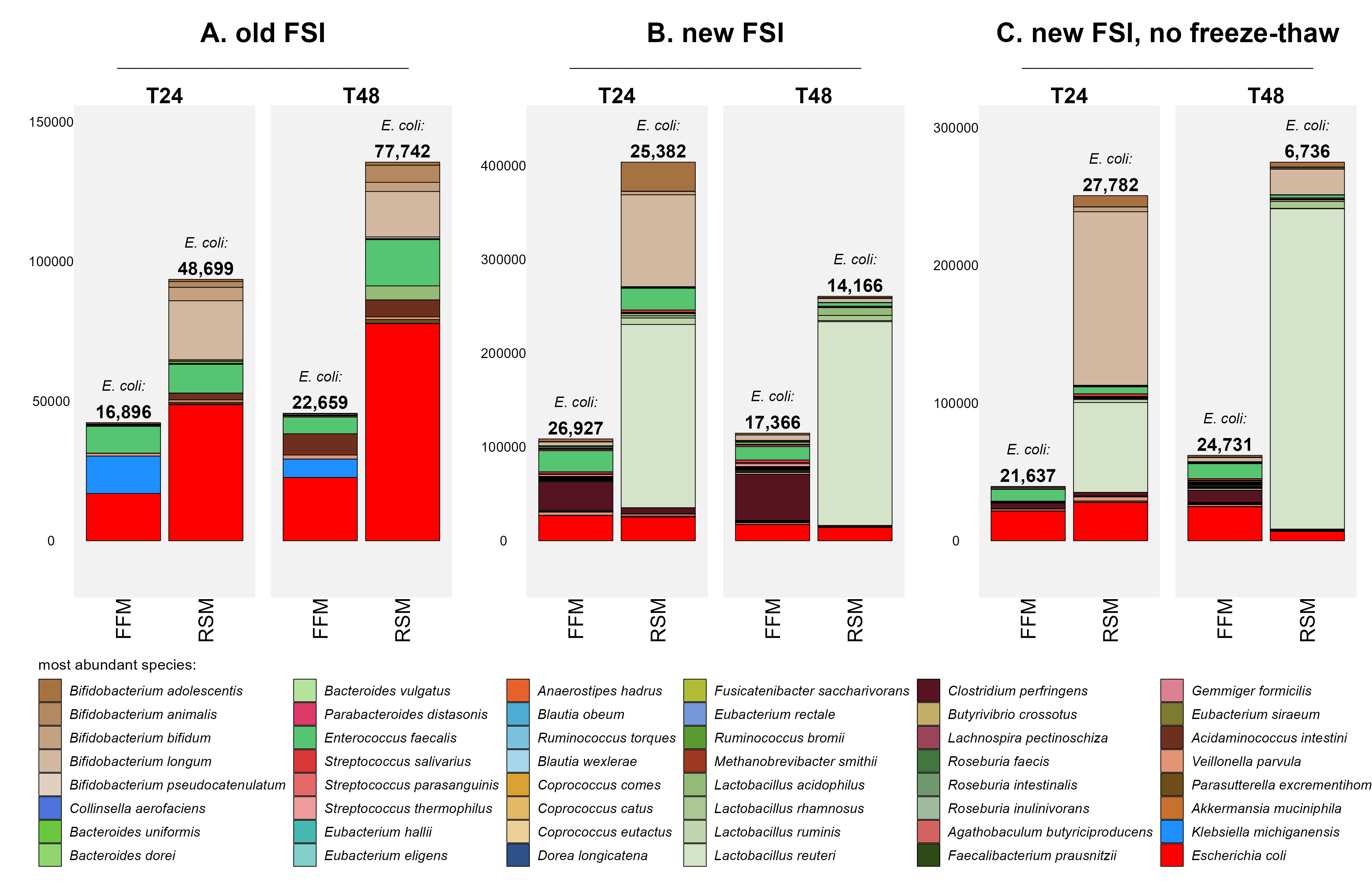

Supplement: Supplementary file 2 [file mmc2.docx]

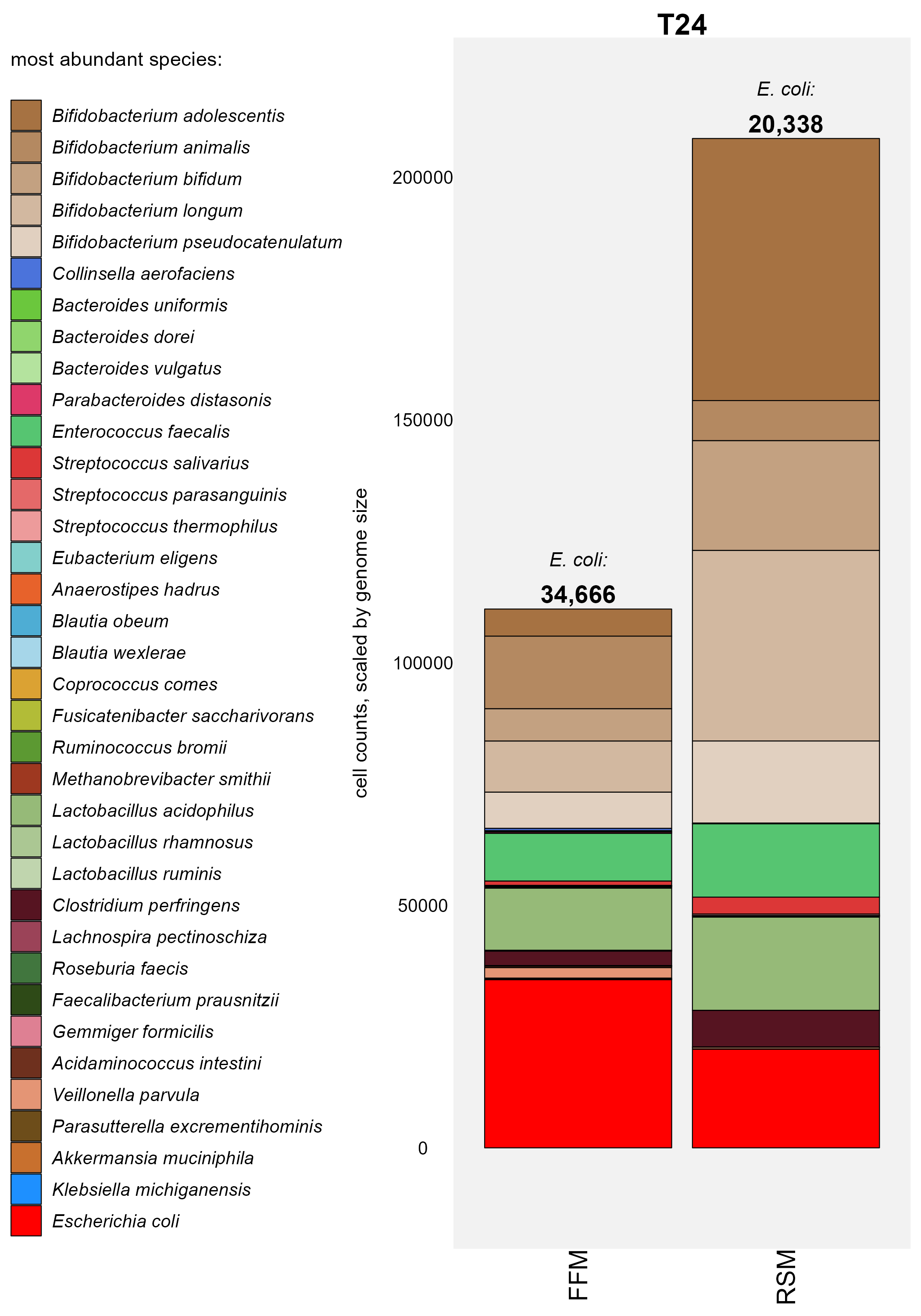

Supplement: Supplementary file 3 [file mmc3.docx]

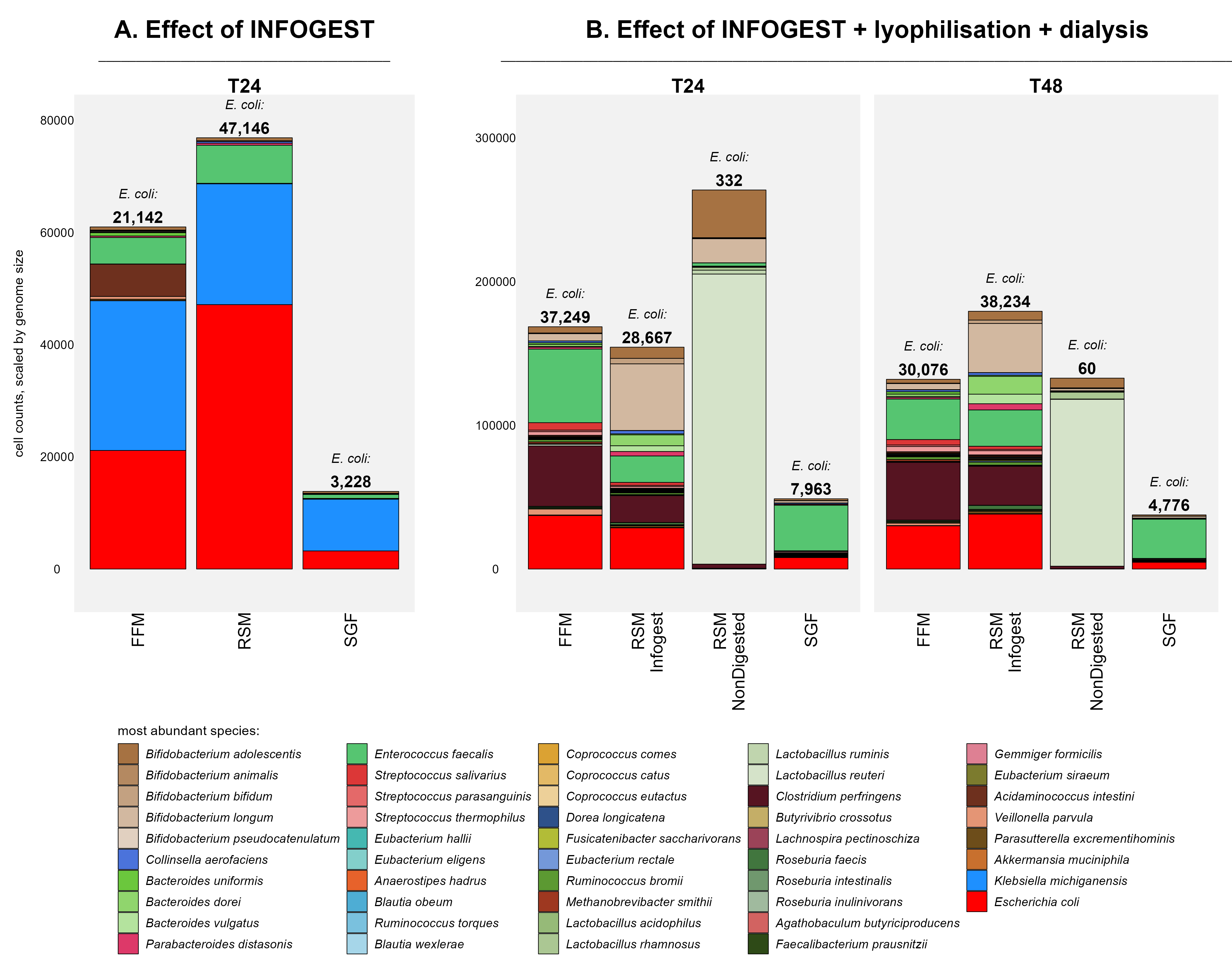

Supplement: Supplementary file 4 [file mmc4.docx]
