# Supplementary material for: Methods to mitigate Escherichia coli blooms in human ex vivo colon model experiments using the high throughput micro-Matrix bioreactor fermentation system
Source: MethodsX. 2023 Oct 6;11:102393. doi: 10.1016/j.mex.2023.102393 (PMC10577065; doi:10.1016/j.mex.2023.102393)
Supplement: Supplementary file 5 [file mmc5.docx]

**Supplementary Section:**

**Methods:**

**Flow cytometry counts of total bacteria in faecal samples from micro-Matrix experiments:**

Total bacterial counts/ml present in the relevant faecal samples from the corresponding micro-Matrix experiments reported herein were determined with a BD Accuri C6 flow cytometer, using the methods described in Mathur et al. 2017 [15] and Field et al. 2019 [16]. Briefly, faecal samples from the relevant micro-Matrix experiments were autoclaved at 121°C for 15 minutes to heat-kill all bacterial cells present in the samples. This was followed by performing serial dilutions in Phosphate Buffered Saline (PBS), followed by staining with 6.68 mM Syto 9 and 40 mM Propidium Iodide from the Live/Dead^TM^ BacLight^TM^ Bacterial viability kit (Invitrogen by Thermo Fisher Scientific L7012) (Life Technologies Corporation, Eugene, Oregon 97402, USA) for 15 min at 37°C, under agitation (400 rpm) in the dark, as described in Mathur et al. 2017 [15]. Quadrant style gating strategies were used to evaluate different subpopulations of bacterial cells as described above and total bacterial counts/ml were calculated. Total *E. coli* cells/ml were calculated by multiplying the percentage of *E. coli* previously determined using the relative abundance percentages described in the main section of the paper, by the total bacterial cell count determined by flow cytometry described here.

**Genome scaling for genome-normalized abundance:**

Metagenomic abundance values were normalized for differences in genome size using a genome scaling approach for the 40 most abundant taxa presented and discussed. Read abundances for these taxa were divided by their respective genome sizes as reported in the NCBI taxonomy database, to give ‘genome-normalized read abundances.’ These values were then used to compute new relative abundance values, and combined with the flow cytometry counts to provide the proportion of *E. coli* counts for the original results reported herein. These normalized values for *E. coli* counts are shown in Supplementary Figures 1-4, corresponding to the original Figures 3-6 respectively in the main section of the paper.

**Figure Legends:**

**Supplementary Figure 1:** Figure corresponding to Figure 3 in the main section of the paper, showing total *E. coli* cell counts based on normalization by a genome-scaling approach in combination with a flow cytometry approach.

**Supplementary Figure 2:** Figure corresponding to Figure 4 in the main section of the paper, showing total *E. coli* cell counts based on normalization by a genome-scaling approach in combination with a flow cytometry approach.

**Supplementary Figure 3:**  Figure corresponding to Figure 5 in the main section of the paper, showing total *E. coli* cell counts based on normalization by a genome-scaling approach in combination with a flow cytometry approach. *Faecal samples for the T6 time point from the corresponding micro-Matrix experiment reported herein were unfortunately not available for flow cytometry analysis as only one aliquot of the relevant samples were taken for T6 and all used up for DNA extractions, as part of the metagenomics study reported in the main section of the paper. Therefore, the T6 time point has been excluded in the normalized stack bar chart for this Figure.

**Supplementary Figure 4:** Figure corresponding to Figure 6 in the main section of the paper, showing total *E. coli* cell counts based on normalization by a genome-scaling approach in combination with a flow cytometry approach.

**References:**

[15] H. Mathur, V. Fallico, P.M. O'Connor, M.C. Rea, P.D. Cotter, C. Hill, R.P. Ross, Insights into the mode of action of the sactibiotic Thuricin CD, Front. Microbiol. 8 (2017). doi: 10.3389/fmicb.2017.00696.

[16] D. Field, T. Blake, H. Mathur, P.M. O' Connor, P.D. Cotter, R.P. Ross, C. Hill, Bioengineering nisin to overcome the nisin resistance protein, Mol. Microbiol. 111 (2019) 717-731. doi: 10.1111/mmi.14183.
